# Diabetic Ketoacidosis After COVID-19 Vaccine in Patients with Type 1 Diabetes Mellitus

**DOI:** 10.5812/ijem-135866

**Published:** 2023-10-08

**Authors:** Fahad Bedaiwi Albedaiwi, Manar Alshammari, Metab Algeffari, Abdulmajeed Alfouzan, Yasmeen Alfouzan, Hassan Siddiq, Omaima Hussein

**Affiliations:** 1 Diabetes and Endocrinology Center, Buraydah, Al-Qassim, Saudi Arabia; 2 Royal Commission Health Services Program, Jubail, Saudi Arabia; 3 Department of Family and Community Medicine, Collage of Medicine, Qassim University, Buraydah, Saudi Arabia; 4 College of Medicine, Qassim University, Buraydah, Al-Qassim, Saudi Arabia

**Keywords:** COVID-19 Vaccine, T1DM, DKA, Bicarbonate

## Abstract

**Background:**

The coronavirus disease 2019 (COVID-19) vaccine is one of the few vaccines that obtained emergency authorization to combat the fatal pandemic. Despite the fact that some available literature addressed its short-term side effects, there are still limitations on its effects on type 1 diabetes mellitus (T1DM).

**Objectives:**

The aim of the present study was to assess the association between COVID-19 vaccination and diabetic ketoacidosis (DKA) among individuals with T1DM. Additionally, the study aimed to determine the effects of the vaccine on glucose control, variability, and risk of hypoglycemia.

**Methods:**

This retrospective study was conducted at King Fahad Specialist Hospital (KFSH) in Qassim Region, Saudi Arabia. Diabetic ketoacidosis cases admitted to the hospital within February 2020 and August 2022 were included in the study based on specific inclusion criteria. Finally, a total of 49 patients were included in statistical analyses.

**Results:**

Out of the 62 patients admitted to the hospital, 49 met the diagnostic criteria for DKA and agreed to participate in the study. The majority of the remaining patients (n = 13) refused to participate, and only a few of them lacked complete documentation. Of the 49 patients who were included in the study, 46 cases had a history of T1DM; nevertheless, 3 patients were newly diagnosed with T1DM. Additionally, among these participants, 16 (32.7%), 19 (38.8%), and 14 (28.6%) patients had mild, moderate, and severe DKA, respectively. There were 27 male (55.1%) and 22 female (44.9%) patients. About 91% of the patients were vaccinated against COVID-19, 30.6% of whom were vaccinated within 29 days of being diagnosed with DKA. The pH and bicarbonate levels were observed to be significantly high among those who were diagnosed with DKA within 29 days of vaccination, with p-values of 0.031 and 0.037, respectively. Similarly, pH and random blood sugar (RBS) were observed to be significantly higher among the vaccinated patients than in the non-vaccinated subjects (P = 0.044 and P = 0.032, respectively).

**Conclusions:**

The study findings revealed that some of the DKA indicators were evident among the vaccinated patients. However, larger-scale and multi-center studies are recommended in order to have more conclusive evidence and generalize the findings.

## 1. Background

The coronavirus disease 2019 (COVID-19) pandemic has had direct and indirect effects on individuals with chronic diseases. According to the World Health Organization (WHO), individuals with chronic diseases are the most vulnerable to severe complications from COVID-19 (1). Therefore, heart disease, diabetes, cancer, chronic obstructive pulmonary disease, chronic kidney disease, and obesity are the conditions that can cause severe illness from COVID-19 (2).

By the start of 2021, some international health authorities announced the successful development of vaccination against COVID-19 and obtained emergency use authorization (3). However, it was unlike the usual practice in medicine, where it takes years to fully develop any vaccine against diseases (4). Consequently, the existing literature primarily focuses on the short-term effects of COVID-19 vaccination, while long-term effects remain a subject for future investigation. Therefore, individuals showed their hesitant behavior in receiving the COVID-19 vaccine due to concerns about potential, yet unexplored, long-term side effects (4, 5). A case-control study was published where patients with chronic diseases were enrolled as cases, and their vaccine acceptance was evaluated in comparison to controls. The study reported the fear of the patients regarding adverse outcomes due to the vaccination (6).

Type 1 diabetes mellitus (T1DM) is a chronic autoimmune disease characterized by increased blood glucose levels due to insulin deficiency that arises from the loss of the pancreatic islet beta cells (7). Type 1 diabetes mellitus normally occurs with an acute clinical course where the patient presents with polyuria, polydipsia, and weight loss (8). Around 8.8% of the adult population worldwide has diabetes mellitus, 10 - 15% of whom are T1DM (9).

Diabetic ketoacidosis (DKA) is an acute metabolic and potentially life-threatening complication of T1DM that contributes to morbidity and mortality and direct and indirect costs of healthcare (10, 11). Previous studies reported that DKA is the leading cause of death among children with T1DM and is associated with increased complications and healthcare expenditure (12, 13). There is a potential chance to reduce the burden of DKA on healthcare outcomes and costs as most DKA cases have already been diagnosed with diabetes. It has been proven that better outpatient care, adherence to self-care, and early detection and treatment of ketones can prevent 50% of DKA-related hospital admissions (14).

Severe hyperglycemia or hyperglycemic emergencies, such as hyperosmolar hyperglycemic syndrome (HHS) and/or DKA, were reported in patients with COVID-19, despite the fact that many of them did not have any history of diabetes (15, 16). Additionally, there were 8 case series that presented with DKA, which was precipitated by COVID-19 infection with positive nasopharyngeal swabs of reverse transcription-polymerase chain reaction (RT-PCR) (17). Among these 8 patients, 5 cases had type 2 diabetes, 1 case had type 1 diabetes, and 2 cases were not diagnosed previously with diabetes. Therefore, COVID-19 infection could precipitate DKA in known diabetic patients or even undiagnosed diabetic patients (17). Another case series reported three cases that presented with acute hyperglycemic emergencies triggered more likely by the COVID-19 vaccine and not by COVID-19 disease. Those patients tested negative by polymerase chain reaction (PCR) for active COVID-19 infection (18). Other case series studies suggested that high glucose levels caused acute DKA among T1DM patients after getting vaccinated against COVID-19 (19, 20).

To the best of our knowledge, no study has been conducted to evaluate the complications of the COVID-19 vaccine in individuals with T1DM. Therefore, this is a strong rationale to conduct the present study to investigate DKA after COVID-19 vaccination in depth.

## 2. Objectives

The objective of this study was to assess if there is any association between COVID-19 vaccination and DKA among individuals with T1DM. Additionally, this study aimed to determine the effect of the COVID-19 vaccine on glucose control, variability, and risk of hypoglycemia.

## 3. Methods

A retrospective cohort study was conducted at King Fahd Specialist Hospital (KFSH), a tertiary care hospital located in the Qassim Region, Saudi Arabia. The region is in the center of the country and has more than 1.2 million population. A total of 62 T1DM patients were selected from all DKA-confirmed cases who were admitted to the hospital within February 2020 and August 2022. Forty-nine subjects met the DKA diagnostic criteria, shown in Table 1. Patients who were younger than 14 years were excluded from the study as they had different local management protocols.

**Table 1.** Diagnostic Criteria for Diabetic Ketoacidosis

Variables	Mild DKA	Moderate DKA	Severe DKA
**Plasma glucose (mg/dL)**	> 250	> 250	> 250
**Plasma glucose (mmol/L)**	> 13.9	> 13.9	> 13.9
**Arterial pH**	7.25 - 7.30	7.00 - 7.24	< 7.00
**Serum bicarbonate (mEq/L)**	15 - 18	10 to < 15	< 10
**Urine ketones**	Positive	Positive	Positive
**Serum ketones - Nitroprusside reaction**	Positive	Positive	Positive
**Anion gap**	> 10	> 12	> 12
**Alteration in sensoria or mental obtundation**	Alert	Alert/drowsy	Stupor/coma

Abbreviation: DKA, diabetic ketoacidosis.

An online central information database is available at this hospital through the hospital information system (HIS) domain, which is secure and restricted within the hospital’s premises. All the study participants’ data and related parameters were part of a previously ordered medically indicated clinical evaluation. Sociodemographic details, medical history, physicians’ notes, laboratory findings, treatment progress notes, and outcomes were retrieved. The laboratory findings included the basic routine laboratory investigations, which include random blood sugar (RBS), fasting blood sugar (FBS), and hemoglobin A1c (HbA1c) levels before, during, and after admission.

Vaccination data included vaccination date, type of vaccine, and the number of doses. These were taken from the “Tawakkalna application”, the official COVID-19 application in the Kingdom of Saudi Arabia. The study was approved by the National Committee of Bioethics (NCBE) of KFSH (approval number: 607-43-2656).

Statistical package for social sciences (SPSS software version 23) was used for data analysis. Descriptive analysis of the data included the mean, standard deviation, frequency, and percentages. The normality of the data was tested using the Shapiro-Wilk test, and the significant results assumed that the data were normally distributed. Additionally, parametric tests were used for inferential data analysis. A two-independent samples *t*-test was used for the comparisons of means in relation to categorical variables with two categories. A paired samples t-test was used for the difference in means of HbA1c before and after DKA. Pearson correlation was computed to study the correlation between variables. All p-values less than 0.05 were considered statistically significant.

## 4. Results

From February 2020 to August 2022, a total of 62 T1DM patients were admitted to King Fahad Specialist Hospital in Buraydah, Qassim Region. Around 49 patients were diagnosed with DKA and admitted to the intensive care unit (ICU). The baseline characteristics of the subjects are shown in Table 2. Various levels of DKA were detected: 28% severe, 38% moderate, and 32.7% mild. Over 33% of the patients were diagnosed with DKA within 29 days of COVID-19 vaccination. Furthermore, 6.1% of the subjects became diabetic after COVID-19 vaccination (Table 2). 

**Table 2.** Frequency Distribution

Variables	No. (%)
**Gender**	
Male	27 (55.1)
Female	22 (44.9)
**DKA severity**	
Mild	16 (32.7)
Moderate	19 (38.8)
Severe	14 (28.6)
DM diagnosed after vaccination	3 (6.1)
DKA within 29 days of vaccination	15 (33.3)
**Vaccinated **	
Yes	45 (91.8)
No	4 (8.2)
**Number of doses**	
1	2 (4.4)
2	20 (44.4)
3	20 (44.4)
4	3 (6.7)
**Age (y)**	20.92 ± 7.2
**A1c pre DKA**	11.66 ± 2.7
**A1c post DKA**	12.22 ± 2.65
**Baseline creatinine (mg/dL)**	0.759 ± 0.38
**C-peptide**	0.17 ± 8.8
**pH**	7.21 ± 0.11
**Bicarbonate**	13.14 ± 4.1
**RBS**	25.32 ± 8.8
**UDS-ketone**	2.29 ± 1.12
**HbA1c**	11.6 ± 2.5

Abbreviation: DKA, diabetic ketoacidosis; DM, diabetes mellitus; RBS, random blood sugar; UDS, unscheduled DNA synthesis; HbA1c, hemoglobin A1c; SD, standard deviation.

Table 3 shows the association between the COVID-19 vaccine and DKA indicators. The pH and bicarbonate averages were significantly high among patients who received the vaccine within 29 days (P = 0.031 and P = 0.037, respectively). However, HbA1c, RBS, or ketone levels did not show a significant difference between the two groups. Furthermore, HbA1c before DKA was significantly lower in individuals who received the COVID-19 vaccine (11.66 [± 2.7] and 12.22 [± 2.65], respectively, P = 0.046). The association between the state of vaccination (yes/no) and the DKA indicators was also tested. It was observed that pH and RBS were significantly higher among vaccinated subjects. The average pH was 7.1 (± 0.13) and 7.2 (± 0.1) among those who did not receive and those who received the vaccine, respectively (P = 0.044). Similarly, the average RBS was 34.3 (± 14.7) in the un-vaccinated patients; however, it was 24.5 (± 7.9) in the vaccinated patients (P = 0.032) (Table 3). 

**Table 3.** Association Between Diabetic Ketoacidosis with Coronavirus Disease 2019 (COVID-19) Vaccination

Variables	DKA Within 29 Days of Vaccination		P-Value
	Yes	No	
**pH**	7.25 (0.09)	7.18 (0.11)	0.031 ^a^
**Bicarbonate**	14.97 (4.1)	12.33 (3.89)	0.037 ^a^
**RBS**	25.1 (8.4)	25.4 (9.1)	0.896
**Ketone**	2.13 (1.12)	2.35 (1.12)	0.532
**HbA1c**	11.01 (2.26)	11.9 (2.54)	0.267
**Variables**	**Vaccinated**		**P-Value**
	**Yes**	**No**	
**pH**	7.21 (0.1)	7.1 (0.13)	0.044 ^a^
**Bicarbonate**	13.4 (4.1)	10.4 (3.8)	0.17
**RBS**	24.5 (7.9)	34.3 (14.7)	0.032 ^a^
**Ketone**	2.24 (1.2)	2.75 (0.5)	0.392
**HbA1c**	11.4 (2.5)	13.3 (1.0)	0.153

Abbreviations: DKA, diabetic ketoacidosis; RBS, random blood sugar; HbA1c, hemoglobin A1c.

^a^ Statistically significant at 0.05 level of significance.

## 5. Discussion

Since the beginning of the COVID-19 pandemic, countries have taken precautionary measures. The vaccine was developed to boost human immunity against the virus and reduce the likelihood of a severe disease outcome (21). However, large-scale testing of the vaccine was not possible to control the disease’s rapid spread and reduce fatalities. As a result, very few studies on post-vaccination complications in individuals with TIDM have been conducted. The purpose of this study was to assess the problems that individuals with TIDM experienced after receiving the COVID-19 vaccine.

According to retrospectively retrieved data, 62 individuals with TIDM were admitted within February 2020 and August 2022, with 49 cases (79%) diagnosed with DKA. Diabetic ketoacidosis severity was moderate to severe in 33 patients (67.4%). About 33% of the patients were diagnosed with DKA within 29 days of COVID-19 vaccination. Diabetic ketoacidosis, HHS, and hyperglycemia post-COVID-19 vaccination have been reported in many case reports (22-25). One hypothesis is that post-vaccination hyperglycemic emergencies could be related to immune-response-mediated hyperglycemia (26).

According to the results of the blood sample analysis, the pH was slightly lower (7.21 ± 0.11) than the normal range. However, bicarbonate levels were observed to be very low among those patients, with an average of 13.14 ± 4.1 mmol/L in the blood, which was very low when compared to the normal range. On the other hand, random blood sugar and unscheduled DNA (UDS)-ketones were observed to be above the normal range. The RBS concentration was 25.32 ± 8.8 mmol/L, and the ketone concentration was 2.29 ± 1.12 mmol/L. Similarly, the HbA1c results were abnormal (11.6% ± 2.5).

Diabetic ketoacidosis criteria include low pH and bicarbonate levels in the presence of ketone bodies in plasma or urine (27). According to Rabbone et al., the risk of severe DKA at the onset of T1DM increased significantly during the severe acute respiratory syndrome coronavirus 2 (SARS-CoV-2) pandemic (28). The relationship between the development of DKA within 29 days of vaccination and blood parameters revealed a significant relationship with both pH and bicarbonate. Those who did not receive the vaccine within 29 days of DKA had significantly lower pH and bicarbonate levels. Nevertheless, the data showed no clear indications that the development of DKA was associated with receiving the vaccine within 29 days. On the other hand, the association between the vaccination status and the DKA indicators showed significant results with pH and RBS. Those who did not receive the vaccine had significantly lower pH and significantly higher RBS than those who received the COVID-19 vaccine.

The researchers have monitored the short-term side effects of COVID-19 vaccination, which include fatigue, pain, fever, chills, headache, nausea, and vomiting (29-31). However, any complications associated with the COVID-19 vaccine that might develop among chronic disease patients have not been extensively studied to date. As a result, chronic disease patients were more hesitant to receive the vaccine than non-chronic disease patients (5, 32). It's important to note that the short-term side effects of the COVID-19 vaccine have been studied, but the long-term effects have not been fully examined, leading to public skepticism regarding the vaccine (5).

In addition to the COVID-19 vaccine, the side effects of the influenza vaccine on diabetic patients were investigated. A type 2 diabetic patient experienced acute hyperglycemia after receiving the influenza vaccine, according to one study (33). In another study conducted by Hulsizer et al., 34 cases of hyperglycemia were reported following influenza vaccination in Texas, USA, between 2018 and 2020 (34). Moreover, there had been 361 reports of hyperglycemia cases with all types of influenza vaccines, according to the Vaccine Adverse Event Reporting System (VAERS) (35). However, very few studies have been conducted and published to report the occurrence of any adverse event associated with the COVID-19 vaccine in patients with chronic diseases.

The current study looked at the occurrence of DKA in T1DM patients after receiving the COVID-19 vaccine. According to the findings of this study, 33% of the patients were diagnosed with diabetic ketoacidosis (DKA) within 29 days of receiving the COVID-19 vaccine, and some of the DKA indicators showed a significant association with COVID-19 vaccination. Since this study was conducted at a single center with a limited number of patients, its findings cannot be considered conclusive or broadly applicable. Moreover, it is recommended that future research with larger, possibly multicenter samples be undertaken to gather more data and achieve conclusive results.

## Data Availability

The dataset presented in the study is available on request from the corresponding author during submission or after publication. The data are not publicly available due to privacy reasons.
